# Application of Genetic Algorithm for Discovery of Core Effective Formulae in TCM Clinical Data

**DOI:** 10.1155/2013/971272

**Published:** 2013-10-30

**Authors:** Ming Yang, Josiah Poon, Shaomo Wang, Lijing Jiao, Simon Poon, Lizhi Cui, Peiqi Chen, Daniel Man-Yuen Sze, Ling Xu

**Affiliations:** ^1^Longhua Hospital Affiliated to Shanghai University of TCM, Shanghai 200032, China; ^2^School of Information Technology, University of Sydney, Sydney, NSW 2006, Australia; ^3^Department of Health Technology and Informatics, The Hong Kong Polytechnic University, Hong Kong

## Abstract

Research on core and effective formulae (CEF) does not only summarize traditional Chinese medicine (TCM) treatment experience, it also helps to reveal the underlying knowledge in the formulation of a TCM prescription. In this paper, CEF discovery from tumor clinical data is discussed. The concepts of confidence, support, and effectiveness of the CEF are defined. Genetic algorithm (GA) is applied to find the CEF from a lung cancer dataset with 595 records from 161 patients. The results had 9 CEF with positive fitness values with 15 distinct herbs. The CEF have all had relative high average confidence and support. A herb-herb network was constructed and it shows that all the herbs in CEF are core herbs. The dataset was divided into CEF group and non-CEF group. The effective proportions of former group are significantly greater than those of latter group. A Synergy index (SI) was defined to evaluate the interaction between two herbs. There were 4 pairs of herbs with high SI values to indicate the synergy between the herbs. All the results agreed with the TCM theory, which demonstrates the feasibility of our approach.

## 1. Introduction

Traditional Chinese medicine (TCM) has been developed and practiced in China for thousands of years, and herbal prescription has played a key role in the medical treatment. A Large number of herbal prescriptions have been recorded over the years where valuable TCM knowledge is hidden. It is urgent and critical to analyze these data so that TCM models can be developed in the modernization of this ancient knowledge. Although TCM is still in practice and more countries consider it as an alternative treatment method [1], the principle of formulating TCM prescription remains unknown. However, it is a daunting task to analyze such a large dataset manually. The methods of knowledge discovery in database (KDD) have been suggested as viable approaches.

KDD allows TCM researchers to find interesting patterns efficiently, and they may direct further laboratory work that leads to discovery [2]. Many successful projects have been reported. For example, Wang et al. [3] illustrated the use of structure equation modeling (SEM) to explore the diagnosis of the suboptimal health status (SHS) and provided evidence for the standardization of TCM patterns. Multilabel learning model [4, 5] was introduced for TCM syndrome identification. Complex network was built for the clinical data mining in TCM [6–8]. 

Generally, KDD research in TCM has been divided into two main categories. The first one attempts to extend our understanding using existing TCM knowledge, while another one attempts to identify core knowledge from existing TCM data, so that each piece of extracted knowledge can be further validated using scientific evidence. This paper belongs to the latter one and, in particular, pays attention to the study on TCM formulae from clinical data.

The efficiency of a formula can be interpreted as a collaboration of its member herbs. It is common to find that most of the prescriptions are of some relatively smaller fixed composition(s) that can be called core formula (CF). Adding herbs into and/or subtracting herbs from CFs are usually carried out in order to realize the personalized treatment. For example, although there are 113 prescriptions in one of the greatest TCM classics, named “Shang Han Lun”, only 8 CFs exist, such as Gui zhi Tang that forms the basis of the formation of Guizhi Jia Gui Tang, Guizhi Xinjia Tang, Gegeng Tang, and Dang gui Si ni Tang [9].

Research on CFs does not only summarize traditional Chinese medicine (TCM) treatment experience, it also helps to reveal the underlying knowledge in the formulation of a TCM prescription. Several computational models were proposed in the past decade to mine the TCM formulae, such as factor analysis [10], the information theory based association rule algorithm [11, 12] or clustering method [13], machine learning models [14], latent tree (LT) models [15], and network analysis [16–20]. These methods can reveal the core herbs and herb-collaboration patterns in TCM prescriptions or uncover the relationship between the herb and symptom, but they seldom concern the related clinical effect. In clinical activities, a number of herbs are combined to form a formula and different formulae are prescribed to different patients, but not all the formulae are effective. It is vital to determine whether a herb combination is effective or not in order to arrive at the valuable formulae. Those core and effective formulae (CEF) are of great interest to TCM practitioners as well as pharmaceutical companies that manufacture medicine using Chinese herbs.

Integrated tumor treatment using Chinese and western medicine is getting standardized in China and has become an important method of prevention and treatment. Many clinical studies [21, 22] considered that TCM is effective and potentially meets the demands of treatment with multitarget therapeutics. Although the current evaluation approach of cancer treatment is still using tumor response and survival as the main indices, TCM concerns the patient as a whole rather than just the tumor; it means that the overall effect should be evaluated instead. Many researchers suggested the use of quality of life (QOL) as a proxy to evaluate the efficacy of TCM treatment [23–25]. To be more specific, it considers the treatment efficacy via the reduction in symptoms severity [26]. For that reason, those herbs combination patterns that are effective in improving symptoms significantly can be regarded as core and effective formulae in TCM tumor clinics.

Therefore, a major goal of this paper is to discuss approaches and strategies for the discovery of core and effective formulae (CEF) in tumor clinical data. Genetic algorithm (GA) was applied, which is a search heuristic that mimics the process of natural evolution [27]. GA generates solutions to optimization problems using techniques inspired by biological evolution, such as inheritance, mutation, selection, and crossover. This is similar to the process of TCM development: prescriptions were created in different herbal combinations for various symptoms, and only the effective prescriptions would make their way into text and records. This is followed by practitioners, who used these effective prescriptions and adapt and create more effective prescriptions. Our previous work [28–30] has proven that, given proper fitness function and search space, GA is suitable for the complex combinatorial optimization in TCM.

This paper is organized as follows. The Materials and Dataset section contains the process of the data acquisition and a description of the data. The Methods section is the methodological part of this paper. It contains definitions related to the assessment of CEF and a description of the genetic algorithm including the definition of fitness function. Complex network is presented in order to address the core herbs analysis in combination of prescriptions, and the analysis of herb-herb interactions is performed in the Results section.

## 2. Materials and Dataset

### 2.1. Data Source

The dataset used in this paper came from the inpatient lung cancer (LC) records of Shanghai Longhua Hospital of TCM. 161 patients with different stages (both early and metastatic stages) of LC only receiving TCM therapy were included. Their prescriptions and symptoms were recorded during February 2010 to August 2012. Traditional Chinese medical herbs were taken as decoction, and fifteen LC symptoms were recorded and they are

1. cough,
2. expectoration,
3. short of breath,
4. chest tightness,
5. chest pain,
6. fatigue,
7. loss of appetite,
8. bloody sputum,
9. dry mouth and throat,
10. fever,
11. spontaneous and night sweating,
12. insomnia,
13. diarrhea,
14. nocturia,
15. five upset hot.

A 4-point response scale (0: not at all, 1: a little, 2: quite a bit, 3: very much) was used to indicate the severity of the symptoms. Since the efficacy of a prescription can only be made known when the patient is met again in the next consultation, hence, to evaluate the efficacy of a prescription and to find the TCM treatment principles, only patients with multiple records (visits) were chosen.

### 2.2. Data Preprocessing and Description

There were 595 transaction records for the 161 patients, which range from 1 to 9 visits, and the average number of transaction records per patient is near 4. Each record has its information of symptoms and the corresponding prescription. The interval of time between two visits was one or two weeks, during which the patient took the same prescription. After excluding those patients who had only one visit, 586 transaction records for the 152 patients were left behind which had a total 230 different herbs being used. In the next stage, the symptom score in each record was calculated as follows:

(1) Symptom  Score=∑i=1mScorei,

where Score_*i*_ represents the score for symptom *i*, and *m* represents the number of symptoms. Symptom change value (SCV) was calculated as the following formula:

(2) $$\begin{array}{c} \text{SCV}=\frac{\text{Previous}\;\text{Symptom}\;\text{Score}-\text{Next}\;\text{Symptom}\;\text{Score}}{\text{Previous}\;\text{Symptom}\;\text{Score}}. \end{array}$$

**Figure 1:**
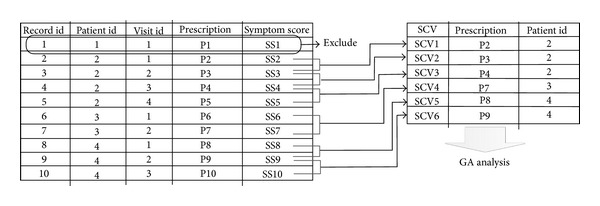
An illustrative example for data format and SCV calculation.

An illustrative example for data format and SCV calculation is shown in Figure 1 where there are 10 transaction records for 4 patients, and the visit range is 1 to 4. The first patient is excluded because of his single visit. Since the symptom score for evaluating the prescription is recorded at the next (following) visit, SCV1 for evaluating prescription “P2” is calculated by “SS2” and “SS3”. In the context of SCV, prescription which belongs to neither single visit nor last visit has its corresponding SCV.

After removing missing values, 419 SCVs for the 150 patients were obtained. According to the TCM theory, the criterion to be effective requires the SCV to be greater than or equal to 30% [60]; in other words, it is a positive outcome and the value is set as 1; otherwise, the outcome is marked as 0.

At the end of this step, 93 out of the 419 records have positive outcome, making it an imbalanced dataset with 22.2% being effective. The statistic information for the number of herbs is shown in Table 1. The top 50 frequently used herbs based on records and patients are shown in Table 2.

## 3. Method

The aim of this paper is to find the core and effective formula (CEF). The measure of effectiveness of a formula helps to determine the efficacy of the herbal interaction in TCM medicine, while the coreness of the prescriptions can help us summarize the TCM treatment principle. The identification of CEF comes from a high dimensional search space of symptoms and herbs; hence, the discovery of CEF can be described as a complicated combinatorial optimization problem. The analytic process of this paper can be described as follows:

1. recognizing and defining the problem,
2. constructing and solving a model for the problem,
3. validating the obtained solutions.

The following sections discuss the different process steps in detail.

### 3.1. Recognizing and Defining the Problem

**Figure 2:**
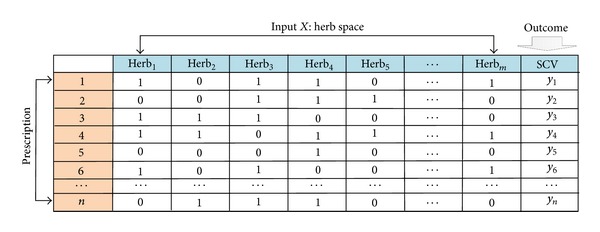
Data format for combinatorial optimization problem of discovery of CEF.

Our problem focuses on how to choose a best combination of herbs. The typical data format is shown in Figure 2. A combinatorial optimization problem *H* = (*Q*, *f*) can be defined by

- a set of herbs *X* = {Herb_1_, Herb_2_,…, Herb_*m*_};
- an outcome variable SCV = {*y*_1_, *y*_2_,…, *y*_*n*_};
- herb domains *D*_1_,…, *D*_*m*_, *D*_*i*_ ∈ {*B*}, where *B* indicates the set of binary values {0,1};
- an objective function *f* to be maximized, where *f* : *D*_1_ × *D*_2_ × ⋯×*D*_*m*_, and *f* ∝ *y*.

The set of possible feasible combinations is

(3) $$\begin{array}{c} Q=\left\{q=\left\{\left(\text{Her}{\text{b}}_{1},v_{1}\right),\left(\text{Her}{\text{b}}_{2},v_{2}\right)\cdots \left(\text{Her}{\text{b}}_{m},v_{m}\right)\right\}\;|\;v_{i}\in D_{i}\right\}, \end{array}$$

where *Q* is a search space which contains all herbs in the data, as each combination of herbs can be seen as a candidate solution. To solve a combinatorial optimization problem of discovery of CEF, we have to find a solution q* ∈ *Q* with maximum objective function value, that is, *f*(q*) ≥ *f*(*q*) for all *q* ∈ *Q*. Before using such a formulation, we have to select the evaluation criterion for CEF. According to the meaning of CEF, the definitions of three heuristics are introduced here.

*(i) Average Confidence of a Core Effective Formula (CEF) Called α*. Let the prescriptions in the dataset be *x*_1_, *x*_2_,…, *x*_*n*_, the number of prescriptions is *n* and the average confidence of a given CEF is

(4) α=∑i=1n(number(CEF∩xi)/number(CEF))n,

where number() is a counting function, ∩ is intersection operator and number(CEF∩*x*_*i*_)/number(CEF) represents the percentage of the common herbs between CEF and *x*_*i*_ with respect to the number of herbs in CEF. The value of *α* is between 0 and 1 inclusive. The larger the value of *α* is, the more representative the given CEF is. When *α* is 1, it implies that every prescription carries all the herbs in the given CEF.

*(ii) Support under Confidences α* *and S*_*α*_. With different confidences, we define support as follows:

(5) Sα=number((number(CEF∩xi)/number(CEF))≥α)n.

The higher the value *S*_*α*_, the higher the occurrence of CEF in the dataset. Let us say we have a CEF with the *S*_0.8_ = 0.25; it means that there are 25% of the prescriptions in the dataset which are composed of at least 80% of the herbs from the given CEF.

*(iii) Effectiveness Value (EV)*. Effectiveness value (EV) is the difference of SCV between two groups. Let the prescription in dataset be *x*_*i*_, *i* = 1,2,…, *n* and their effectiveness (outcome variable) *y*_*i*_, *i* = 1,2,…, *n*. If x* is a CEF and ((number(x*∩*x*_*i*_)/number(x*))/*n* ≥ *α*, it means that the confidence of *x*_*i*_ is greater than or equal to *α*_CEF_; when *α* is greater, it indicates that the proportion of herbs from x* being used in *x*_*i*_ is higher than the average confidence *α*. In other words, *x*_*i*_ is an application of x*. Let *x*_1_* denote the group of all the *x*_*i*_, namely, CEF group, otherwise, *x*_0_*, namely, non-CEF group. The effectiveness value (EV) is defined as

(6) $$\begin{array}{c} \text{EV}=\frac{{\overset{-}{Y}}_{x_{1}^{*}}-{\overset{-}{Y}}_{x_{0}^{*}}}{{\text{std}}_{x_{1}^{*}x_{0}^{*}}}. \end{array}$$

If *y*_*i*_ is continuous, then ${\overset{-}{Y}}_{x_{1}^{*}}$ and ${\overset{-}{Y}}_{x_{0}^{*}}$ represent the average effectiveness of the group of *x*_1_* and *x*_0_*, respectively. If the *y*_*i*_ is binary, then ${\overset{-}{Y}}_{x_{1}^{*}}$ and ${\overset{-}{Y}}_{x_{0}^{*}}$ represent the effective proportion of the group of *x*_1_* and *x*_0_*, respectively. std_*x*_1_*x_0_*_ represents the joint standard deviation. The bigger the value EV is, the better the effectiveness is. Furthermore, we should consider the minimum number of herbs contained in x*.

### 3.2. Constructing and Solving a Model for the Problem

**Figure 3:**
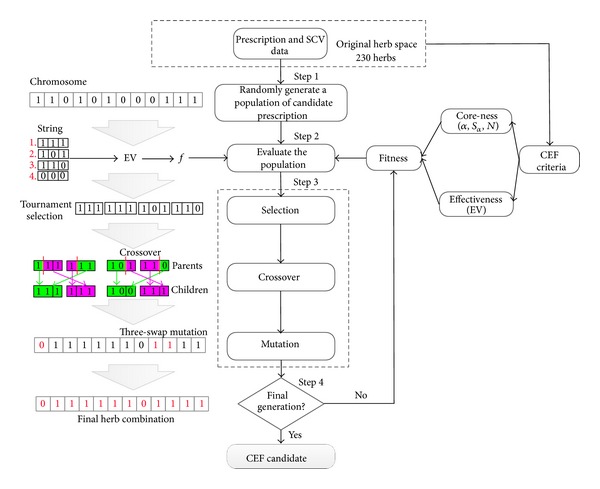
Flow chart of GA and explanations of the sequence of GA steps for the discovery of CEF.

To construct and solve a model for combinatorial optimization is a difficult task: in general, we start with a realistic but possible solution, and then execute iterative optimization. As a computational model of evolutionary processes, GA not only has the ability to solve combinatorial optimization problems that are nonparametric, in contrast to most other algorithms that find one solution at a time, but also it has the strength to find multiple pareto optimum solutions in parallel at the same time. This is compatible with TCM treatment that multiple formulae are applicable to a set of symptoms, that is, it is an equifinality. The concept of equifinality refers to many alternative ways of attaining the same objective. Using the previous definitions in Section 3.1, the sequence of steps of GA for the application of the discovery of CEF is shown in Figure 3.

**Step 1 (*encoding and initial population*)**

The herb combination to be optimized is represented by a chromosome whereby each herb is encoded in a binary string called gene according to the original herb space. Since there were 230 distinct herbs, the chromosome was made up of a string of 230 binary characters, with the value of “0” and “1” to describe a prescription. A population, which consisted of a given number of chromosomes, was initially created by randomly assigning “1” to all genes with probability *P*_*i*_. The value “1” in a gene meant that the corresponding herb was used in this prescription. Otherwise, the herb was not used in this prescription.

**Step 2 (*the design of fitness function (objective function*))**

A crucial point in using GA is the design of fitness function, which determines what a GA should optimize. The goal of this study is to find CEF, which is a small subset of herbs that are frequently used and most significant for effectiveness. Fitness was measured by two criteria of CEF, one is coreness that is represented by *α*, *S*_*α*_, and *N* (minimum number of herbs contained in CEF), and the other is effectiveness that is represented by EV. *N* is typically decided by TCM theory, while the determination of *S*_*α*_ depends on how representative and frequently used the required CEF are. An important characteristic of GA is the way it deals with infeasible solutions (unsatisfactory CEF). The offspring might be potentially infeasible when recombining solutions. The most general and simple way is to reject infeasible solutions. Therefore, penalizing infeasible solutions in the fitness function that measures the qualification of a solution is more appropriate, which was presented in our research. Hence, the fitness function, *f*, is defined as follows:

(7) f={EV,if  Sα≥Sαset,  N≥NsetEV−R,else,

where *S*_*α*set_ and *N*_set_ are the predefined values of the support under a certain confidence and the minimum number of the herb contained in x* (mentioned above). *R* is a penalty constant, which is used to penalize the infeasible solutions. Thus, the evaluation of fitness started with the randomly generating prescription that was composed of all the presence of herb whose gene was coded as “1.” Then the prescription's coreness and effectiveness were evaluated by *S*_*α*_, *N*, and EV. Finally, the fitness was measured by the fitness function *f*. In the context of *f*, those prescriptions whose coreness meet the requirement with high EV will have the higher probability to survive.

**Step 3 (*design of the GA operator*)**

After evolving the fitness of the population, the chromosomes were selected by means of the tournament selection, which involved running several “tournaments” among a few chromosomes chosen at random from the population. The winner of each tournament (the one with the best fitness) was more likely selected. Then children chromosomes were created from parent chromosomes by multipoint crossover operator. After that, the chromosomes were mutated with a three-way swap of three randomly chosen genes in a permutation, which could lead to new chromosomes in the searching space. Sometimes, this may lead to new and better results. Mathematically, using crossing over is helpful to find a local optimal solution, and mutations can help to discover new and better optima.

**Step 4 (*terminal condition*)**

GA is an iterative search method, which will approach the optimized region but may not arrive at the optimized solution. So a terminal condition is needed. Here, we terminated GA process after a predefined number of generations. The chromosomes of the last generation with the highest value of *f* were considered to be the CEF candidates.

### 3.3. Validating the Obtained Solutions

After finding optimal or near-optimal solutions (prescriptions), we had to evaluate them. Based on the meaning of CEF, solutions were evaluated on both coreness and effectiveness. In this study, the measurements of confidence and support are proxy to the coreness property of a formula. The definitions *α* and *S*_*α*_ which denote the confidence and support of a solution were used to evaluate the coreness. Generally speaking, when *S*_*α*_ has to be calculated, *α* should have a minimum value of 0.7 according to the TCM expert practitioner, otherwise, it may undermine the representative property of CEF. The greater these two values are, the better the coreness of a solution is, that is also to say, the constituent herbs are more widely used in the prescriptions and the formula is more frequently used. As for the evaluation of effectiveness, the dataset can be divided into two groups, namely, the CEF group and non-CEF group. By the definition mentioned in Section 3.1, in CEF group, all the records' prescriptions carried more than preset *α* proportion of herbs in the specific solution, while the prescriptions in the non-CEF group did not. Then *Z*-test for the difference between two effective proportions (EP) was carried out at a 5% significance level (*P* < 0.05).

## 4. Results

### 4.1. GA Results

**Figure 4:**
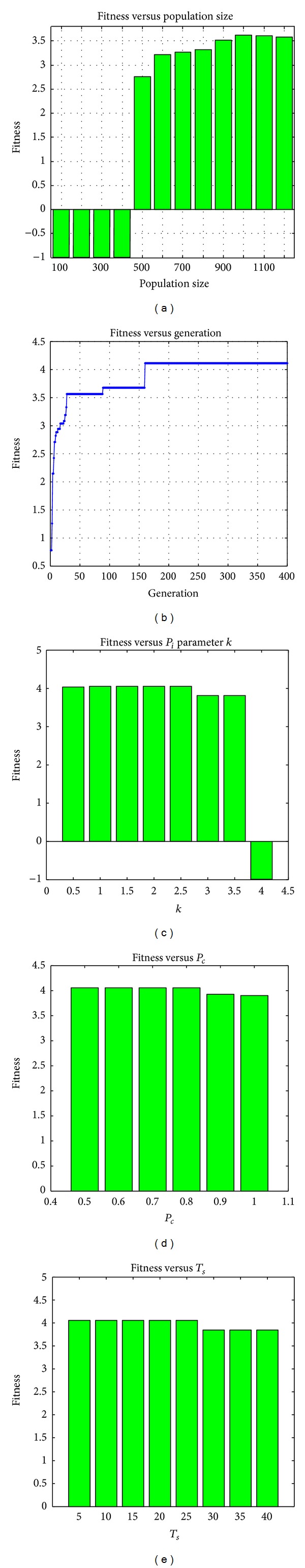
Parameter selection in GA.

The results in the following discussion were averaged over three executions using the same parameters. To compare effect of changing the parameters on GA efficiency and results, we needed to fix all the parameters of fitness function. The fixed configuration used for fitness function is being described here: *R* (the penalty constant) was set to 200, *N*_set_ was set to 8, *α* was set to 1, and its corresponding *S*_1_ was set to 2%. First, we investigated the effect of population size. Here, generation was initially set to 100 and the other basic parameters of the GA followed the default. The population sizes were chosen from 100 to 1200 with the step size of 100. We have summarized the results in Figure 4. The figure only compares the average of best fitness values.  Figure 4(a) shows that fitness values are acceptable when the population sizes are greater than 400, and fitness values are better (exceed 3.5) when the population sizes are greater than 900. While bigger population needs more time for the algorithm to run, in this study, we use a population size of 1000 in our experiments. Second, we fixed population size at the optimal value, then generation number was chosen to be 400. It can be seen from Figure 4(b) that generation number has positive effect on fitness value found. When generation number reaches 160, the fitness value is the best and remains unchanged. We, therefore, select generation number of 200 in this paper. Similarly, we compared the effect of the other parameters of GA, in turn. We consider initial herb selection probability (*P*_*i*_,  *P*_*i*_ = k*N_set_/230)  *k* ∈ {0.5,1, 1.5,2, 2.5,3, 3.5,4}, crossover probability *P*_*c*_ ∈ {0.5,0.6,0.7,0.8,0.9,1.0}, and tournament selection size *T*_*s*_ ∈ {5,10,15,20,25,30,35,40}. Figures 4(c)~4(e) show the results. We can see that fitness values are acceptable when *k* is smaller than 4. When *k* equals 4, the initial number of herb selection is close to the maximum number of herb in prescription, which leads to filtering out the most feasible solutions. So we set *k* to 1 in this paper. While *P*_*c*_ and *T*_*s*_ are insignificant with respect to fitness value, we set them to the default values.  Table 3 lists the parameters of the GA for the experiments in this paper. 

**Figure 5:**
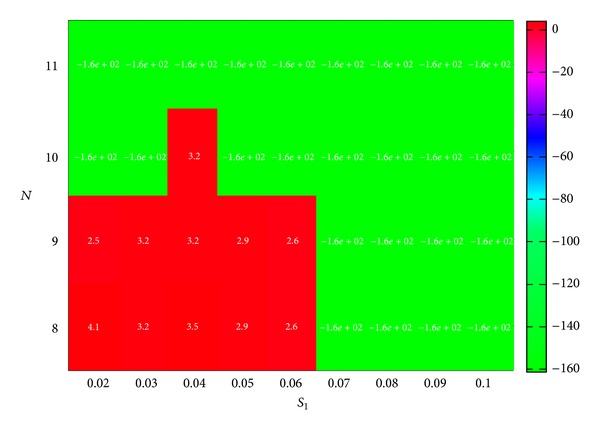
Fitness value by GA with different *N* and *S*_1_ combination.

As for the parameters of fitness function, in order to get CEF with the highest confidence, *α* was set to 1. The other parameters were set to the following values: *R* (the penalty constant) is set to 200 and *N*_set_ were given in the range of 8 to 11, while *S*_1_ began from 2% and stepped up by 1% in each increment until it reached 10%. A heat map in Figure 5 shows the sensitivity of fitness values in relation to the different setup of *S*_*α*set_ and *N*_set_. We can see that fitness values are mostly positive with values of *N*_set_ being 8 or 9, while *S*_1_ is in the range of 2~6%. After removing duplicates, there were 9 CEF which are solutions with positive fitness values. These 9 CEF are composed of 15 distinct herbs (Tables 4 and 5). We summarize the traditional indications and effects of cancer treatment for these herbs in Table 4. According to the clinical experiences and the literature reports, these herbs as well as their extracts or isolated compounds can exert their anticancer effects in several ways: (a) they can enhance immunity and body resistance; (b) they have antiproliferative activity in cancer cells; (c) they can improve quality of life and prolong the life span of the patients.  Table 5 shows that the maximum number of herbs in a CEF is 11, which is much smaller than the average number of herbs in a transaction in the dataset. There are a few herbs existing that are common across 6 CEF, such as AF, AO, AR, DS, PC, PT, RB, and TP. According to TCM terms, these common herbs are related to nourishing *Yin*, regulating *Qi*, and strengthening the spleen function, which are generally consistent to the TCM principle in LC treatment.

### 4.2. Evaluation

#### 4.2.1. Coreness

Herb-herb network was constructed using a cooccurrence frequency-based method. The degree value of one node (herb) was defined as the number of other nodes (herbs) that it connects to; it is a simple but an important property of any complex network. A node has a more significant role to play if it has a higher degree value. The importance of a herb was studied according to its degree value and frequency in the dataset. These values were sorted into descending order and shown in Table 6. Among the 230 herbs in the dataset, the 15 herbs that make up CEF are all ranked in the top 50 in terms of degree and frequency based on both records and patients. In other words, it is a good indication that these 15 herbs in CEF are core herbs.

Average confidence of the prescription (*α*) and support under the different *α* confidence (*S*_*α*_) were calculated in order to evaluate the coreness of CEF. In order to evaluate the correlation within individual, patient-based support (PBS) was also calculated for each CEF (Table 7). The values of *α* and *S*_*α*_ are all relatively high. In particular, the second CEF (CEF2) has its *α* value that exceeds 0.7, which means that the prescriptions in dataset are consistently composed of more than 70% herbs from the CEF2. The values under *S*_0.7_ of both CEF8 and CEF9 exceed 0.5, which means that there are more than 50% of the prescriptions that are composed of 70% or more herbs from these two CEF. As for PBS, a CEF is not valuable for its small PBS when it is concentratedly used for the minority. Results show that all PBS are larger than the corresponding *S*_1_ (record-based support), which indicates no concentrated use on patient level for CEF.

#### 4.2.2. Effectiveness

To test the effectiveness of CEF, the dataset was divided into two groups, namely, the CEF group and non-CEF group. In this study, *α* was set to 1. In other words, all the prescriptions in CEF group carried all the herbs of the specific CEF, while the prescriptions in the non-CEF group did not have a full set of herbs from CEF. The *Z*-test for the difference between two effective proportions (EP) was performed for each CEF.  Table 8 shows that EP of all the CEF groups are significantly better than the non-CEF group.

Sampling is a simple and well-known method for parameter studies and robustness evaluations [61]. To test the robustness of effectiveness in this study, leave one (patient) out analysis was performed. After removing one patient from the original data, effectiveness of CEF was remeasured for the remaining patients. This was repeated such that each patient in the data was removed once. EP and *P* value of *Z*-test were calculated for each CEF each time. Results are shown in Table 9. Then *P* value was transformed into −log⁡⁡(*P*), where −log⁡⁡(*P*) was larger than 1.301 indicating that *P* value was smaller than 0.05. It can be seen in Table 9 that there is little change in EP of both groups from the original to the perturbed and all −log⁡⁡(*P*) exceed 1.33, which shows good robustness for the effectiveness evaluation with a small perturbation in sample (patient level) space.

### 4.3. Assumption Analysis

There were 9 CEF and 15 core herbs generated from the GA process. Since the number of distinct herbs from the overall CEF was relatively small, we want to find out whether a CEF consisting of these 15 core herbs exists or not, if so, check its effectiveness. It was found that such a combination of herbs was in the dataset. Its coreness and effectiveness were evaluated (Table 10). Although *α* and EP values were relatively high and may be acceptable, *S*_1_ value was only 0.009, which meant there were only 4 records covering this combination. Its value is too small to be considered as a core formula, but it is still worthwhile to carry out clinical trial in the future because of its higher effectiveness.

### 4.4. Analysis of Herb-Herb Interactions in CEF

**Figure 6:**
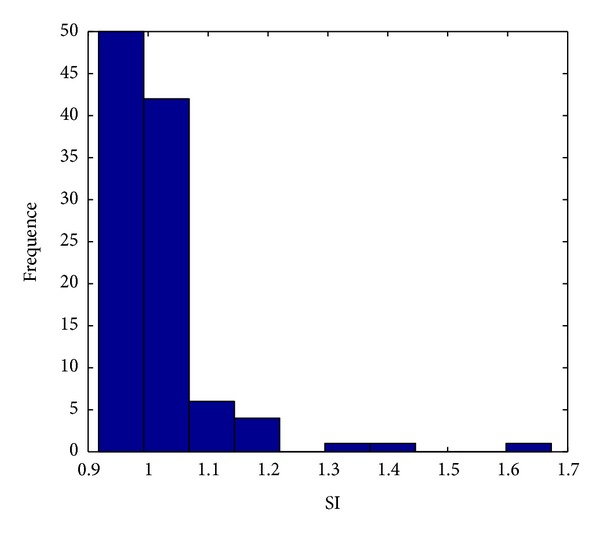
Distribution of SI.

A herb combination is chosen to promote desirable herb-herb interaction; the efficacy of a TCM formula comes from the synergistic effects of its constituent herb pairs. Therefore, practitioners are interested to identify the potential interacting herbs from a prescription. Based on the previous work [7] of the analysis of herb-herb interactions in CEF, the synergy index (SI) was calculated for each herb pair in CEF as follows:

(8) SI=E11E01∨E10,

where *E*_11_ denotes the EP value of cooccurring of the two herbs and *E*_01_ or *E*_10_ is the EP value of each one used without the other herb, while ∨ denotes a maximum function, that is max⁡⁡(*E*_01_, *E*_10_). When SI is equal to 1, it indicates no real advantage of putting the two herbs together. When SI is greater than 1, it shows potential synergy. When SI is getting a larger value, it indicates a synergistic interaction between the two herbs in the pair.  Figure 6 shows the distribution of SI of all core herb pairs. Although most SIs are closed to 1, the distribution skews more to the positive side (greater than 1), which indicates the existence of some potential synergies. All the SIs values are greater than 0.9, which imply no obvious antagonistic effect among the core herb pairs. Permutation test [7] was performed to test the significance of SI by permuting the outcome variable 2000 times. As a result, 4 significantly synergistic effects of core herb pairs were obtained (Table 11). Table 11 shows that most of these pairs were related to the functions of regulating *Qi* to promote diuresis and eliminating dampness to eliminate phlegmon according to TCM theory.

## 5. Discussion

Prescription for a diagnosis is a complicated and flexible procedure that integrates the knowledge of TCM theory. TCM practitioners put heavy emphasis on individualities when prescribing formulae in clinical practices. This is very different from the modern western medical therapies that usually comply with a common and operational clinical guideline. Revealing the regularity in prescriptions is an important step to reveal the underpinning TCM theory. It has generated much research interest to discover the regularity from the TCM prescriptions. Although computational models have been applied to reveal the core herbs and herb-collaboration patterns, not much effort has been expended to study their effectiveness. This is a critical and important research to discover these hidden patterns that are core and effective herbal formula.

As for the discovery of CEF, it can be described as a complicated combinatorial optimization problem mathematically, which is concerned with the efficient combination of herbs to meet requirement. The purpose of this study is to set the stage and give an outline of properties of optimization problems that are relevant for discovery of CEF in TCM. We described the process of how to define this problem model that could be solved by GA method. In brief, analytic process consisted of recognizing and defining problems, constructing and solving models, and evaluating solutions. Furthermore, we looked at important properties of CEF, which could be used as the validation criteria. For CEF, there are two key questions to be answered. One is how to evaluate the coreness of a TCM formula and the other is the assessment of its clinical effectiveness.

In this study, the measurements of confidence and support are proxy to the coreness property of a formula; the greater these two values are, the more widely the constituent herbs are used in the prescriptions and the more frequently the formula is used. The definitions *α* and *S*_*α*_ denote the confidence and support of a given CEF, respectively. It is quite common for a TCM practitioner to pick a subset of formulae, which are CEF, as templates, and personalize them for a patient. Upon the selected template(s), the practitioner can add or remove or replace herbs. The confidence value (*α*) well explains the flexible usage of the CEF and the personalized adaptation in action. 

Regarding the assessment of clinical effectiveness, the primary outcome measurement in our study was to quantify information related to the symptoms changes in a cancer treatment. In an internal panel meeting of TCM cancer experts, the most common LC symptoms were identified and they were consistent with the literature [26, 62]. Our results show that the total improvement proportion in symptoms was only 22.19%, which indicated a great challenge for the LC treatment for the TCM practitioners. Of course, it makes no sense that the frequently used herb combinations (CEF candidates) do not have high efficacy.

GA has the ability to solve combinatorial optimization problems, which was reported by the literature [63–65]. A basic GA has the following implementation steps. First, the feature values are encoded into chromosomes to form the initial population. Second, calculate the fitness of every chromosome using the defined fitness function. Thirdly, according to the fitness values, genetic operators are applied to select chromosomes to form a new population. This process is repeated until a certain condition is satisfied. In our previous work [29], GA has successfully helped us to find a meaningful relationship between herbs and symptoms after designing a proper fitness function. Therefore, it is our belief that the usefulness of GA for other combinatorial optimization problems in TCM cannot be fairly assessed on the basis of its performance on the discovery of herb-symptom relationship alone.

In this study, we gave an outline description of the way in which a genetic algorithm worked. While a crucial point in using GA is the design of the fitness function, which determines what a GA should optimize. In this study, we designed the fitness function based on two evaluation criteria of CEF, one is coreness which is represented by confidence and support defined in the present paper, the other is effectiveness which is evaluated by the statistic difference in effective proportion between CEF group and non-CEF group. The proposed fitness function is flexible and suitable for both binary and continuous outcome. To apply a penalty constant *R* in the fitness function is the strategy of removal of unsatisfactory CEF. This constant could be set to a value greater than the maximum value of EV to identify the CEF that meet the requirement; that is, a CEF would be dropped if the fitness value was negative (not meeting expectation), otherwise it would be kept.

Parameter tuning is always a challenging task for GA. The GA toolbox for Matlab developed by the University of Sheffield was used in these experiments. We implement and run the algorithm using different configurations and compared results. Results show that some parameters need careful selection of settings like population size, generation, and *P*_*i*_. Others are insignificant with respect to fitness value and can follow the default. As for fitness function, the additional key parameters are *N* and *S*_*α*_ in our approach. In this study, *α* was set to 1 and *S*_1_ began from 2%. When *S*_1_ was high or *N* get large, there were not many CEF with positive values. A small but reasonable number of CEF were reported after proper values were set for *S*_*α*_ and *N*.

In particular, for multiple records data, which can be also regarded as longitudinal data, there are three types of correlation effects: (1) correlation between variables (herbs), (2) correlation within individual (patient), and (3) correlation between individuals (patients).

As for the research of TCM formula, the first one can be seen as the herb-herb relationship; such relationships are meaningful patterns of herb combination, which provokes many researchers to develop methods to uncover the underlying rules. For this purpose, support- and confidence-based association rules algorithms are generally introduced. Motivated by the idea of association algorithm, we presented the support- and confidence-based criteria (*α* and *S*_*α*_) in order to evaluate the coreness of herb combination. It is found that when *α* is equal to 1, *S*_*α*_ is effectively the concept of support as commonly used in the association mining algorithm, such as Apriori algorithm [66–69].

It is hard to tackle the second correlation, which may undermine the evaluation of herb combination. For example, when one CEF is used for only one patient who visits frequently, although its support may be relatively high because of its large number of times for visit, such CEF is meaningless. However this disadvantage can be reduced by choosing a large sample size. Hence, individual- (patient) based support analysis could be helpful to identify the correlation within patient. In this paper, we gave support based on the patient for the analysis and carried out robustness analysis of CEF's effectiveness by the leave one (patient) out method. Results showed no concentrated use on patient level for CEF and good robustness also implied the stability for the effectiveness evaluation with a small perturbation in sample (patient level) space, which meant that correlation within patient level in this study did not undermine our evaluation on the effectiveness of CEF and our sample size was appropriate for discovering the reliable solutions.

The last correlation is related to the individual's factors, such as age, gender, pathology, family history, pulmonary function, and TCM syndrome. In order to reveal the relationship between patient pattern and CEF, another mathematical pattern recognition model needs to be established, which will be in our future work.

A total of 9 CEF were reported with good *core* property and high effectiveness. In the calculation of the EP value of single use of each core herb, the maximum value was 31.3% that was significantly lower than any combinations (CEF). These results highlighted the advantages and rationality of the combined use of herbs in TCM and were also meaningful for further experimental researches.

In the theory of TCM, deficiency is the important cause and pathogenesis during the occurrence and development of tumor. Lack of vital *Qi* and deficiency of both *Qi* and spleen can lead to a series of pathological changes, such as *Qi* stagnation, blood stasis, dampness, and phlegm, and eventually lead to the tumor [7, 70–73]. For that reason, the TCM treatment to lung cancer is guided by strengthening body resistance, including benefiting *Qi* and nourishing *Yin*, and it is also supplemented by eliminating pathogens including dissipating phlegm, promoting blood circulation to dispel blood stasis, and detoxification[70, 73]. The prescription can be divided into two major parts [74].

*Strengthening [73]*. The emphasis of the treatment is invigorating spleen and kidney. Si jun zi decoction which is made up of Codonopsis, AO, PC, and Licorice is a classic prescription to invigorate spleen and replenish *Qi*. RB characterized by spleen, lung, and kidney can nourish spleen and kidney and benefit lung for promoting production of fluid. All the 9 CEF contain the medication intentions above-mentioned, for example, Codonopsis and AR which are both characterized by spleen and lung can benefit *Qi* for promoting production of fluid, and AR also has the function of invigorating *Qi* to consolidate the superficies and expelling pathogens by strengthening vital *Qi* and expelling pus. As a kind of common Chinese herb, AR is often used to strengthen resistance and to remove toxic substance instead of Codonopsis.

*Eliminating Pathogens [73]*. The common function of PT and CSE is eliminating dampness and phlegm. AO combined with PC can invigorate spleen for eliminating dampness to enhance the effectiveness of softening and resolving hard mass. CS and AF can regulate *Qi*-flowing for promoting blood circulation to remove blood stasis. DS and HO can clear heat toxicity.

All these functions complement each other in order to achieve the effect of a treat for a disease by looking into both its root cause and symptoms. What is more, as a consumptive disease of lung cancer, the digestive function would decline over time, so RS, MA, TP, and CD help to resolve food stagnation and promoting herb absorption.

One interesting observation is the similarity among the CEF; this can help understand underlying TCM therapeutic principles for LC. Since it is fairly common for the doctors in the same hospital to use similar sets of herbs for the same disease (LC), it is necessary and beneficial to compare the results of CEF with an LC dataset from another hospital. It is also worthwhile to observe what CEF are discovered if a larger dataset with higher supports is used.

The herb-herb interactions in CEF were also studied and reported. Four herb pairs with high and significant SI values indicate that they were synergistic. Some of them are present in classic TCM formulae. For example, PT and TP are in Ban xia Chen pi Tang, which contribute to the relieving of cough and reducing sputum.

Therefore, all the results conformed with TCM theory, which indicated the feasibility and validity of the proposal. However, dosages not considered in this work, which are a key aspect in CEF, should be taken as the future work. GA is capable of representing its chromosomes in real numbers, and a reformulation of the fitness function can accommodate this change. A mathematical model of dose-effect needs to be defined. This may increase the complexity of the definition of the fitness functions, but the valuable results will make the effort worthwhile.

## 6. Conclusions

After the confidence, support, and effectiveness values related to a CEF were introduced, GA was used to discover the CEF from a TCM cancer clinical dataset. Results indicated that GA is suitable for the discovery of CEF that can be interpreted from the TCM principles. This is just an attempt and exploration of data mining to discover CEF from TCM clinical data. More work is still required to explore the strength, limitation, and appropriateness of the measures if they are relevant to other types of diseases.

## Figures and Tables

**Table 1:** Statistic information for number of herbs.

	Per record	Per patient	Average number per patient per record
Minimum	9	9	9
Average	23	29	22
Maximum	36	73	33

**Table 2:** Top 50 frequently used herbs.

Rank	Record based		Patient based	
	Herb	Frequency	Herb	Frequency
1	Chinese sage herb	395	Chinese sage herb	147
2	Doederlein's spikemoss herb	393	Doederlein's spikemoss herb	146
3	Akebia fruit	359	Akebia fruit	133
4	Herba *oldenlandiae *	332	Herba *oldenlandiae *	129
5	*Atractylis ovata *	321	*Atractylis ovata *	127
6	Rice-grain sprout	268	Astragalus root	116
7	Malt	268	*Pachyma cocos *	107
8	*Pachyma cocos *	266	Chicken gizzard membrane	107
9	Astragalus root	263	Rice-grain sprout	106
10	Chicken's gizzard-membrane	252	Malt	106
11	Common selfheal spike	239	Common selfheal spike	104
12	Rhizoma batatatis	235	Rhizoma batatatis	97
13	Coix seed	223	Coix seed	96
14	Tangerine peel	195	Coastal glehnia root	86
15	Coastal glehnia root	195	Oysters	85
16	Oysters	183	Tangerine peel	80
17	Rhizoma amorphophalli	172	Pericarpium trichosanthis	79
18	Pericarpium trichosanthis	162	Asparagus cochinchinensis	72
19	Asparagus cochinchinensis	158	Rhizoma amorphophalli	68
20	Edible tulip	139	Edible tulip	64
21	*Crataegus pinnatifida *	133	*Crataegus pinnatifida *	62
22	*Ophiopogon japonicus *	122	Tatarian aster root and rhizome	58
23	Chinese date	120	Shorthorned epimedium herb	58
24	*Glycyrrhiza uralensis *	119	Pilose asiabell root	58
25	Pilose asiabell root	119	*Ophiopogon Japonicus *	57
26	Tatarian aster root and rhizome	112	*Glycyrrhiza uralensis *	55
27	Chinese taxillus herb	111	Chinese taxillus herb	53
28	Shorthorned epimedium herb	109	Chinese date	53
29	*Pinellia* tuber	99	*Pinellia* tuber	51
30	Baikal skullcap root	98	Heartleaf *houttuynia* herb	48
31	Suberect spatholobus stem	97	Suberect spatholobus stem	46
32	Heartleaf *houttuynia* herb	95	Glossy privet fruit	46
33	Glossy privet fruit	91	Baikal skullcap root	45
34	Noble *dendrobium* stem herb	89	Balloon flower root	43
35	Chekiang fritillary bulb	82	Chekiang fritillary bulb	42
36	Paris polyphylla smith	79	*Eucommia* bark	40
37	Almond	78	Paris polyphylla smith	40
38	*Eucommia* bark	76	Barbary wolfberry fruit	40
39	Balloon flower root	75	Almond	40
40	Barbary wolfberry fruit	73	Noble *Dendrobium* stem herb	39
41	*Pyrrosia* leaf	65	Cherokee rose fruit	35
42	Cherokee rose fruit	63	Chinese dodder seed	32
43	Reed rhizome	57	*Pyrrosia* leaf	31
44	Toad skin	57	Reed rhizome	30
45	Radix *semiaquilegia *	55	Toad skin	30
46	Fingered citron fruit	55	Common macrocarpa fruit	29
47	Chinese dodder seed	54	Immature bitter orange	28
48	Common macrocarpa fruit	53	Radix *Semiaquilegia *	25
49	Dragon's bones	51	Radish seed	24
50	Immature bitter orange	51	Dragon's bones	24

**Table 3:** Parameters for GA.

Parameter	Value
Population size	1000
Initial herb selection probability (*P*_*i*_)	*N*_set_/230
Crossover probability (*P*_*c*_)	0.7
Tournament selection size (*T*_*s*_)	15
Generation	200

**Table 4:** Traditional indications and biological effects of herbs.

Herb	Abbreviation	Traditional indications^#^	Effects of cancer treatment	Reference
Astragalus root	AR	To reinforce *Qi* and invigorate the function of the spleen.	Immune stimulating effect. Improving quality of life for patients with nonsmall cell lung cancer.	[31, 32]
Akebia fruit	AF	To regulate *Qi*, to promote blood circulation and relieve pain, and to cause diuresis.	Popularly used for primary liver cancer treatment in China.	[33]
Atractylis ovata	AO	To invigorate the function of the spleen and replenish *Qi* and to eliminate dampness by causing diuresis.	Antiangiogenic activity. Inhibiting the growth of B16 cancer cells.	[34–38]
Chinese date	CD	To tonify the spleen, replenish *Qi* and to nourish blood.	Antiproliferative activity in human breast cancer cells.	[39]
Chinese sage herb	CS	To remove toxic heat and blood stasis and relieve pain.	Antiangiogenic activity.	[40]
Coix seed	CSE	To transform dampness and promote water metabolism, to strengthen the spleen, and to clear heat and eliminate pus.	Affecting cellular pathways in neoplasia: to inhibit NFkappaB and protein kinase C signaling.	[41]
Doederlein's spikemoss herb	DS	To remove toxic heat and dampness and to promote blood circulation and remove blood stasis.	Antiproliferative activity in three types of human cancer cells in vitro.	[42]
Herba *Oldenlandiae *	HO	To eliminate heat and toxic material, to promote blood circulation and remove blood stasis, and to clear dampness heat.	Antiproliferative activity in eight cancer cell lines. Strengthening the patient's resistance.	[43–45]
Malt	MA	To invigorate the function of the spleen, to regulate the function of the stomach, and to promote the flow of milk.	Proliferative function of colonic epithelial cells.	[46–49]
*Pachyma cocos *	PC	To cause diuresis, to invigorate the spleen function, and to calm the mind.	Inhibiting the growth of nonsmall cell lung cancer cells.	[50, 51]
*Pyrrosia* leaf	PL	To induce diuresis, relieve dysuria, remove heat, and arrest bleeding.	Its active components: isomangiferin has capability of inhibiting virus replication within cells, and fumaric acid has chemopreventive potential for tobacco-nitrosamine-induced lung tumors.	[52, 53]
*Pinellia* tuber	PT	To remove damp and phlegm, to relieve nausea and vomiting, and to eliminate stuffiness in the chest and the epigastrium.	Antiproliferative activity in five cancer cell lines in vitro.	[54, 55]
Rhizoma batatatis	RB	To replenish the spleen and stomach, to promote fluid secretion, and to benefit the lung.	Inhibiting the cancer cell line of melanoma B16 and Lewis lung cancer in mice in vivo.	[56]
Rice-grain sprout	RS	To promote digestion, invigorate the function of the spleen, and improve appetite.	Popularly used for strengthening function of the spleen and the stomach during cancer treatment in China.	[57]
Tangerine peel	TP	To regulate the flow of *Qi*, to invigorate the spleen function, to eliminate damp, and to resolve phlegm.	Antioxidative and anti-inflammatory functions. Antiproliferative activity in human gastric cancer cells.	[58, 59]

^#^Information is queried from TCM-ID database (http://bidd.nus.edu.sg/group/TCMsite/).

**Table 5:** CEF obtained by GA.

No.	Number of herbs	Composition														
		AR	AF	AO	CD	CS	CSE	DS	HO	MA	PC	PL	PT	RB	RS	TP
1	10		X	X			X			X	X	X	X	X	X	X
2	9	X	X	X		X		X	X	X			X	X		
3	8	X	X	X							X		X	X	X	X
4	9	X	X	X				X	X		X		X	X		X
5	8		X	X		X		X			X		X	X		X
6	10			X	X	X	X	X	X		X		X	X		X
7	9	X	X	X		X		X		X			X	X		X
8	11	X	X	X		X		X	X		X		X	X	X	X
9	10	X	X	X				X		X	X		X	X	X	X
		6	8	9	1	5	2	7	4	4	7	1	9	9	4	8

**Table 6:** Core herb identification.

Herb	Degree	Degree rank	Record based		Patient based	
			Frequency	Frequency rank	Frequency	Frequency rank
DS	225	1	393	2	146	2
CS	225	2	395	1	147	1
AF	223	3	359	3	133	3
AO	220	4	321	5	127	5
HO	219	5	332	4	129	4
PC	207	6	266	8	107	7
AR	198	9	263	9	116	6
CSE	197	10	223	13	96	13
RB	194	11	235	12	97	12
RS	191	12	268	6	106	9
MA	191	13	268	7	106	10
TP	184	16	195	14	80	16
CD	158	30	120	23	53	28
PT	152	34	99	29	51	29
PL	127	47	65	41	31	43

**Table 7:** Confidence and support of CEF.

No.	α	*S*_0.7_	*S*_0.8_	*S*_0.9_	*S*_1_	PBS
1	0.549	0.396	0.248	0.084	0.021	0.040
2	0.707	0.499	0.239	0.041	0.041	0.067
3	0.598	0.375	0.198	0.043	0.043	0.073
4	0.653	0.356	0.191	0.050	0.050	0.073
5	0.675	0.489	0.294	0.084	0.084	0.120
6	0.616	0.461	0.265	0.122	0.036	0.060
7	0.670	0.406	0.196	0.041	0.041	0.067
8	0.678	0.501	0.310	0.167	0.041	0.067
9	0.637	0.511	0.332	0.181	0.041	0.067

**Table 8:** *Z*-test for the difference of EP for CEF.

No.	EP of non-CEF group	EP of CEF group	*P* value
1	0.210	0.778	0.000
2	0.206	0.588	0.002
3	0.204	0.611	0.000
4	0.206	0.524	0.004
5	0.203	0.429	0.009
6	0.210	0.533	0.013
7	0.206	0.588	0.002
8	0.206	0.588	0.002
9	0.206	0.588	0.002

**Table 9:** Leave one (patient) out analysis to test the robustness of effectiveness (total 150 times).

No.	EP of non-CEF group		EP of CEF group		−log (*P*)	
	Mean	Range	Mean	Range	Mean	Range
1	0.209	[0.204, 0.213]	0.778	[0.750, 0.857]	4.215	[3.308, 5.867]
2	0.206	[0.201, 0.210]	0.589	[0.563, 0.643]	2.764	[2.314, 3.194]
3	0.204	[0.199, 0.208]	0.612	[0.588, 0.667]	3.273	[2.791, 3.779]
4	0.206	[0.201, 0.209]	0.524	[0.500, 0.579]	2.360	[1.990, 2.936]
5	0.203	[0.197, 0.206]	0.429	[0.412, 0.455]	2.036	[1.757, 2.337]
6	0.210	[0.205, 0.214]	0.533	[0.500, 0.583]	1.859	[1.335, 2.160]
7	0.206	[0.201, 0.210]	0.589	[0.563, 0.643]	2.764	[2.314, 3.194]
8	0.206	[0.201, 0.210]	0.589	[0.563, 0.643]	2.764	[2.314, 3.194]
9	0.206	[0.201, 0.210]	0.589	[0.563, 0.643]	2.764	[2.314, 3.194]

**Table 10:** *Core*-ness and effectiveness evaluation of 15 core herbs combination.

α	*S*_1_	EP of non-CEF group	EP of CEF group	*P* value
0.605	0.009	0.217	0.750	0.014

**Table 11:** Analysis of herb-herb interactions in CEF.

No.	Herb pair		SI	*P* value
1	PL	PT	1.673	0.004
2	CD	PT	1.419	0.012
3	CSE	PL	1.363	0.028
4	PT	TP	1.077	0.025
